# Mortality and functional outcomes after a spontaneous subarachnoid haemorrhage: A retrospective multicentre cross-sectional study in Kenya

**DOI:** 10.1371/journal.pone.0217832

**Published:** 2019-06-12

**Authors:** Peter Waweru, Samwel Maina Gatimu

**Affiliations:** 1 Neurosurgery Department, M.P Shah Hospital, Nairobi, Kenya; 2 School of Nursing and Midwifery, Aga Khan University, Nairobi, Kenya; Universitatsklinikum Freiburg, GERMANY

## Abstract

**Introduction:**

Despite a reduction in poor outcomes in recent decades, spontaneous subarachnoid haemorrhage (SAH) remains associated with severe disability and high mortality rates. The exact extent of these outcomes is however unknown in Africa. This study aimed to determine the mortality and functional outcomes of patients with SAH in Kenya.

**Methods:**

We conducted a retrospective multicentre cross-sectional study involving patients admitted with SAH to three referral hospitals in Nairobi. All patients with a confirmed (primary) discharge diagnosis of first-time SAH between January 2009 and November 2017 were included (n = 158). Patients who had prior head trauma or cerebrovascular disease (n = 53) were excluded. Telephone interviews were conducted with surviving patients or their next of kin to assess out-of-hospital outcomes (including functional outcomes) based on modified Rankin Scale (mRS) scores. Chi-square and Fisher’s exact tests were used to assess associations between mortality and functional outcomes and sample characteristics.

**Results:**

Of the 158 patients sampled, 38 (24.1%) died in hospital and 42 (26.6%) died within 1 month. In total, 87 patients were discharged home and followed-up in this study, of which 72 reported favourable functional outcomes (mRS ≤2). This represented 45.6% of all patients who presented alive, pointing to high numbers of unfavourable outcomes post SAH in Kenya.

**Conclusions:**

Mortality following SAH remains high in Kenya. Patients who survive the initial ictus tend to do well after treatment, despite resource constraints.

**Limitations:**

The study findings should be interpreted with caution because of unavoidable limitations in the primary data. These include its retrospective nature, the high number of patients lost to follow up, missing records and diagnoses, and/or possible miscoding of cases.

## Introduction

Spontaneous subarachnoid haemorrhage (SAH) is associated with severe disability and high mortality rates; despite a reduction in case fatality rates over the last four decades [1]. Recent studies have reported mortality rates from 25% at pre-hospital [2] to 18% in hospital [3]. Mortality also remains high after discharge from hospital, with rates of 40% within 30 days [4], 50% within 1 year [5] and an elevated long-term mortality [6]. Only half of patients with SAH who get to the hospital return to their previous level of functioning. The remainder are plagued by numerous complications, from gross neurological deficits to subtle cognitive and neurobehavioural difficulties that hinder normal functioning and lower quality of life [7,8].

SAH in sub-Saharan Africa accounts for approximately 10–16% of all stroke subtypes [9,10]. Autopsy studies show that it contributes to 2% of all unexplained non-violent deaths and nearly 16% of all cardiovascular causes of deaths in Kenya [11]. This is despite only a third of patients in some stroke studies having radiological confirmation [10] and an even smaller number having angiographies [12]. As a result, SAH in sub-Saharan Africa is diagnosed late [13]. This, coupled with delayed presentation [14], limited neurosurgical services and treatment options [13], and high cost of care, mean that a majority of patients are untreated, and a good number expire [15]. After a protracted hospital stay being conservatively managed, others end up with poor neurological outcomes [13]. Although reports in Kenya point to a high case fatality [15–17], the extent to which spontaneous subarachnoid haemorrhage patients survive and function after discharge, have not been studied. Therefore, we aimed to determine the mortality and functional outcomes of patients with SAH in Kenya.

## Methods

### Study design and setting

We conducted a retrospective multicentre cross-sectional study to clarify the outcomes of patients with SAH in Kenya. This study was conducted in one public and two private referral hospitals located in Nairobi city. These three hospitals are some of the largest referral hospitals in the country and offer both outpatient and inpatient services, including neurosurgical and neuro-intensive care.

### Sample and data collection

**Fig 1:**
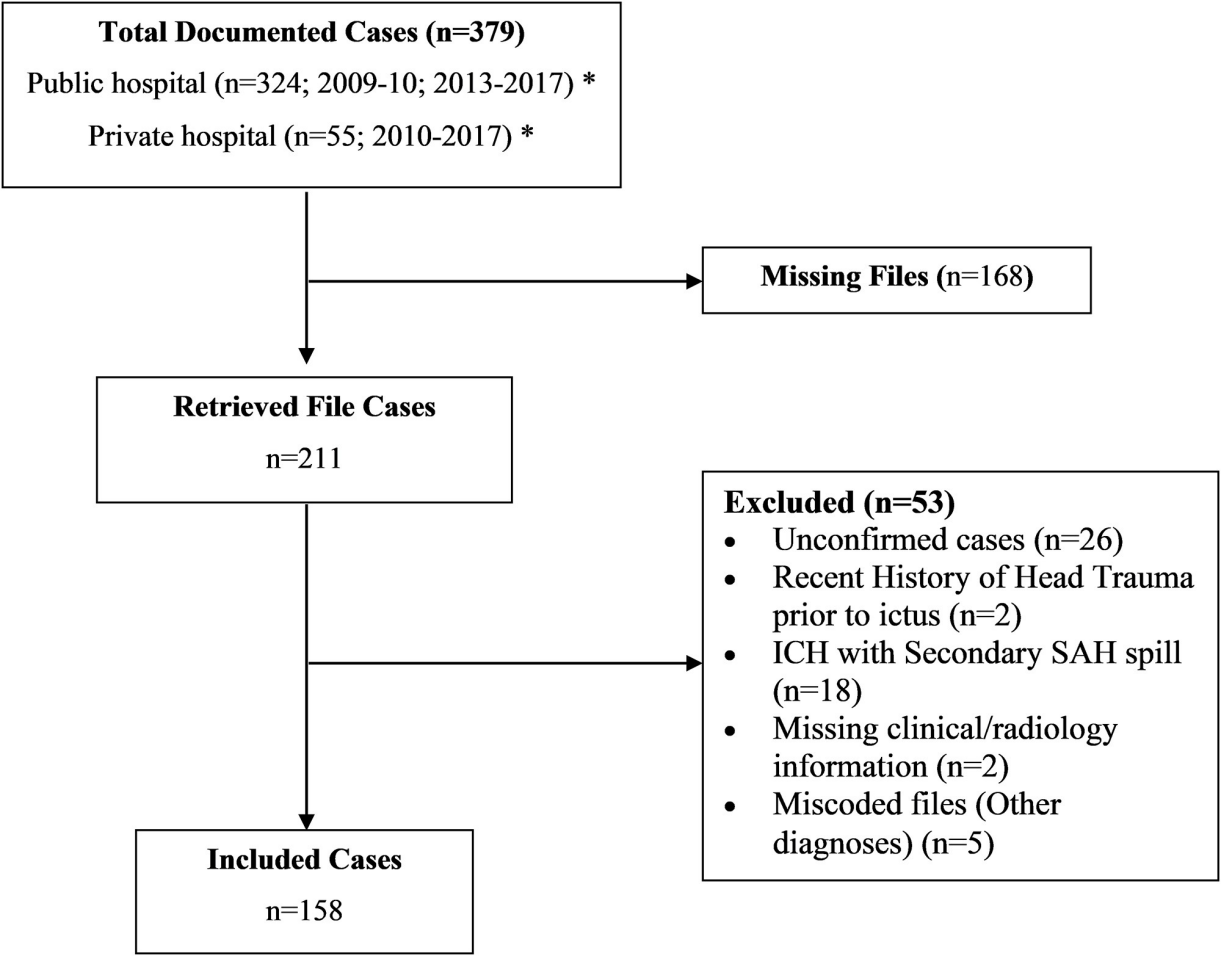
Study sample. *Files from the public hospital for the years 2011 and 2012 were untraceable. Similarly, files in the private hospitals were untraceable for the year 2009. **ICH**: intracerebral haemorrhage; **SAH**: subarachnoid haemorrhage.

All adult and child patients with a primary confirmed discharge diagnosis of first-time SAH (International Classification of Diseases, tenth revision: I60) and admitted to the selected hospitals between January 2009 and November 2017 were included. We excluded all cases of intracerebral haemorrhage with subarachnoid extension and those with a documented history of head trauma. We identified 379 cases of SAH (324 in public and 55 in private hospitals). Data were retrieved for 158 cases because 168 files were missing, and 53 cases were excluded. Hospital records for 2009 in the private hospitals and 2011–2012 in the public hospital were lost; as such, these cases could not be assessed (Fig 1).

The principal investigator and four trained research assistants retrieved available files from the hospital medical records departments. Data on patients’ sociodemographic characteristics, contact information, medical history, clinical presenting features and modes of treatment were extracted from hospital records and patients’ files using a standardised data extraction tool. Additional data on mortality and functional outcomes were collected from patients or their next of kin. This information was collected via telephone interviews. All participating patients/next of kin provided verbal informed consent, and the interviews were conducted by the principal investigator using an encrypted telephone to ensure confidentiality.

### Case definition

SAH was defined as a suggestive history (including but not limited to) a history of abrupt onset of a severe headache or unconsciousness with or without signs of meningeal irritation or focal neurological signs supported by computed tomography, lumbar puncture or necropsy evidence of SAH.

### Measures

The main outcomes for this study were in-hospital and 1-month post-discharge mortality rates. The secondary outcome was functional outcomes measured using modified Rankin Scale (mRS) scores; mRS scores ≤2 were classified as favourable and mRS scores of 3–6 were considered unfavourable. Exposure measures included: demographic characteristics; presenting clinical, radiological and angiography features; and treatment modes.

### Statistical analysis

STATA version 13 was used for the statistical analyses, with the significance level set at p<0.05. Descriptive statistics were used to describe the sample characteristics, mortality and functional outcomes. Associations between in-hospital mortality and functional outcomes and sample characteristics were assessed using chi-square and Fisher’s exact tests.

### Ethics

Ethical approval was obtained from the Kenyatta National Hospital, University of Nairobi Ethics Review Board (Ref: KNH–UON/P319/06/2017). Permission to conduct the study was granted by all the three participating hospitals before the study started. Informed verbal consent was provided by all participating patients or their next of kin before telephone interviews, and an encrypted telephone line used for the interviews. All collected data were de-identified, and serial numbers were used for data collection.

## Results

### Sample characteristics

In total, 158 patients with SAH were included in this study. A majority were female (n = 91, 57.6%), the mean age was 48.6 (standard deviation [SD], 15.9; Range: 10–93) years and most were from the public hospital (n = 121, 76.6%). There was an almost equal number of old (≥50 years) and young (<50 years) patients (49% vs. 51%), and of those with/without hypertension (52% vs. 48%). Patients presented with acute headache (86.5%), depressed consciousness (53.2%), neck pain or stiffness (43.6%), seizures (21.8%) and focal neurological deficits (13.5%). The modal admission Glasgow coma scale (GCS) score was 13–15 (68.35%) and the average systolic blood pressure was 148.5 mmHg (SD, 29.6 mmHg). Most patients were discharged with a GCS of 13–15 (95.3%). There were no significant differences in patient characteristics between private and public hospitals except for alcohol use which was more among patients in the public hospital (p = 0.006) and admission systolic blood pressure which was higher among patients in the private hospital (p = 0.043) (Table 1).

**Table 1:** Sample characteristics by hospital type ^a^.

	Variables	Private n (%)	Public n (%)	Total n (%)	p-value
**Demographic and clinical characteristics**	**Sex**				
	Male	20 (54.1)	47 (38.8)	67 (42.4)	0.101
	Female	17 (45.9)	74 (61.2)	91 (57.6)	
	**Age**				
	<50 years	19 (52.8)	61 (50.4)	80 (51.0)	0.803
	≥50 years	17 (47.2)	60 (49.6)	77 (49.0)	
	History of hypertension ^b^ (n = 153)	14 (37.8)	65 (53.7)	79 (50.0)	0.166
	Tobacco use (n = 127) ^b^	4 (15.4)	14 (13.9)	18 (14.2)	0.763
	Alcohol use (n = 126) ^b^	12 (46.2)	20 (20.0)	32 (25.4)	0.006*
	Depressed consciousness ^b^	19 (51.4)	64 (53.8)	83 (53.2)	0.796
	Seizures ^b^	8 (21.6)	26 (21.8)	34 (21.8)	0.977
	Headache ^b^	32 (86.5)	103 (86.5)	135 (86.5)	0.992
	Neck pain or stiffness ^b^	11 (29.7)	57 (47.9)	68 (43.6)	0.052
	Focal neurological deficits ^b^	21 (13.5)	21 (13.5)	21 (13.5)	0.275
	Ictus to presentation, days ^c^	3 (1–5)	4 (2–7)	4 (2–7)	0.126
	Admission SBP (n = 156), mean (SD)	152 (34.1)	147.5 (28.1)	148.5 (29.6)	0.043*
	**Admission GCS (n = 155)**				
	3–8	3 (8.3)	15 (12.6)	18 (11.6)	0.111	
	9–12	3 (8.3)	26 (21.8)	29 (18.7)		
	13–15	30 (83.3)	78 (65.6)	108 (69.7)		
**Radiological features**	**Modified Fisher’s score (n = 38)**				
	1	6 (16.2)	0 (0.0)	6 (3.8)	
	2	3 (8.1)	2 (1.7)	5 (3.2)	0.088
	3	5 (13.5)	8 (6.6)	13 (8.2)	
	4	7 (18.9)	7 (5.8)	14 (8.9)	
	Missing score	16 (43.2)	104 (86.0)	120 (76.0)	
	Hydrocephalus (n = 90)	11 (57.9)	18 (25.4)	29 (32.2)	
**Angiography**	**Angiogram status (n = 91)**				
	Positive ^d^	23 (62.2)	37 (30.6)	60 (38.0)	0.215	
	Negative	9 (24.3)	22 (18.2)	31 (19.6)		
	Not Done	5 (13.5)	62 (51.2)	67 (42.4)		
	**Angiogram type (n = 91)**				
	Computed tomography	26 (81.3)	15 (25.4)	41 (45.1)	<0.001*	
	Magnetic resonance	1 (3.1)	6 (10.2)	7 (7.7)		
	Digital subtraction	5 (15.6)	38 (64.4)	43 (47.2)		
	**Aneurysm location (n = 56)** ^e^				
	Anterior (ACA/AcoA)	11 (47.8)	15 (39.5)	24 (42.9)	0.832	
	Middle (MCA)	6 (26.1)	8 (21.1)	11 (19.6)		
	Internal (ICA/PcoA)	4 (17.4)	7 (18.4)	11 (19.6)		
	Posterior (PCA/VA)	0 (0.0)	2 (5.3)	1 (0.02)		
	Multiple	2 (8.7)	6 (15.8)	9 (16.1)		
	**Aneurysm size (n = 56)**				
	Small	6 (26.1)	1 (3.0)	7 (12.5)	0.350	
	Medium	7 (30.4)	1 (3.0)	8 (14.3)		
	Large	0 (0.0)	1 (3.0)	1 (1.8)		
	Missing	10 (43.5)	30 (90.9)	40 (71.4)	
**Management**	**Mode of treatment**				
	Conservative	17 (46.0)	108 (89.3)	125 (79.1)	<0.001*	
	Clip	14 (37.8)	13 (10.7)	27 (17.1)		
	Referral (for coiling)	6 (16.2)	0 (0.0)	6 (3.8)		
	Days from ictus to clipping	5.5 (2–9)	19 (11–58)	9 (4–20.5)	0.006*
	Length of hospital stay	9 (7–15)	11 (5–21)	10.5 (5–21)	0.250
	**Discharge GCS**				
	3–8	2 (6.4)	1 (1.3)	3 (2.8)	0.234	
	9–12	0 (0.0)	2 (2.6)	2 (1.9)		
	13–15	29 (93.6)	73 (96.1)	102 (95.3)		

**ACA**: anterior cerebral artery; **AcoA**: anterior communicating artery; **GCS**: Glasgow Coma Scale; **ICA**: internal carotid artery; **MCA**: middle cerebral artery; **PCA**: posterior cerebral artery; **PcoA**: posterior communicating artery; SAH: subarachnoid haemorrhage; **SD**: standard deviation; **SBP**: systolic blood pressure; **VA**: vertebral artery;

* statistically significant at p = 0.05

^a^ Detailed table with the missing values is available S1 Table

^b^ Includes only the YES responses [Detailed results are available in S1 Table]

^c^ Median (range/interquartile range)

^d^ Includes 4 arteriovenous malformations

^e^ Only patients with angiogram positive are included

### SAH types and management

A total of 91 (57.6%) patients, 52 (49.2%) in public and 32 (84.2%) in private hospitals underwent a cerebral angiogram. Forty-three patients (47.2%) had a digital subtraction angiogram, 41 (45.1%) had a computed tomography angiogram and the remainder had a magnetic resonance angiogram. Sixty patients (38%) and 31 (19.6%) had a positive and negative angiogram results. Aneurysm was identified in 61.5% (n = 56) of the 91 patients who underwent a cerebral angiogram, with most being in the anterior circulation (n = 24, 42.9%) and of medium (n = 8, 50%) or small sizes (n = 7, 44%). Four patients, two males and two females below 50 years of age of those with positive angiogram had arteriovenous malformations (Tables 1 and 2). Sixty-seven (42.4%) of all SAH patients, a majority of who were female, aged ≥50 years and seen in public hospital did not undergo cerebral angiogram.

**Table 2:** In-hospital and functional outcomes by sample characteristics.

Variables	In-hospital mortality (n = 158)			Functional outcomes (n = 87)^c^		
	Dead (n, %)	Alive (n, %)	p-value	Favourable (n, %)	Unfavourable (n, %)	p-value
**Hospital type**						
Public	33 (86.8)	88 (73.3)	0.09	52 (81.3)	12 (18.7)	0.53
Private	5 (13.2)	32 (26.7)		20 (87.0)	3 (13.0)	
**Sex**						
Male	18 (47.4)	49 (40.8)	0.48	35 (48.6)	3 (20.0)	0.05*
Female	20 (52.6)	71 (59.2)		37 (51.4)	12 (80.0)	
**Age**						
<50 years	13 (35.1)	67 (55.8)	0.03	40 (55.6)	5 (33.3)	0.16
≥50 years	24 (64.9)	53 (44.2)		32 (44.4)	10 (66.7)	
Admission SBP, mmHg [mean (SD)]	149.5 (28.0)	148.2 (30.2)	0.81	142.7 (27.0)	164.1 (26.4)	*
**Admission GCS**						
3–8	14 (37.8)	4 (3.4)	<0.01*	2 (2.9)	0 (0.0)	<0.01*
9–12	12 (32.4)	17 (14.4)		4 (5.7)	5 (33.3)	
13–15	11 (29.7)	97 (82.2)		64 (91.4)	10 (66.7)	
**Hydrocephalus**	9 (47.4)	20 (28.2)	0.11	9 (20.0)	5 (50.0)	0.05*
**Angiogram Status**						
Positive (n = 56) ^a^	7 (18.4)	49 (42.2)	0.48	27 (38.6)	8 (53.3)	0.03*
Negative (n = 31)	1 (2.7)	30 (25.9)		20 (28.6)	0 (0)	
Angiogram Not Done (n = 67)	30 (78.9)	37 (31.9)		23 (32.8)	7 (46.7)	
**Aneurysm size (n = 16)**						
Small	1 (33.3)	6 (46.2)	>0.99	2 (28.6)	2 (66.7)	0.67
Medium	2 (66.7)	6 (46.2)		4 (57.1)	1 (33.3)	
Large	0 (0.0)	1 (7.7)		1 (14.3)	0 (0.0)	
**Mode of treatment**						
Conservative	35(92.1)	90 (75.0)	0.10	53 (73.6)	12 (80.0)	0.29
Clip	3(7.9)	24 (20.0)		18 (25.0)	2 (13.3)	
Referral (for coiling)	0 (0)	6 (5.0)		1 (1.4)	1 (6.7)	
**Length of hospital stay, days** ^b^	8 (4–21)	12 (8–17)	0.03*	11.5 (8–16.5)	13 (8–47)	0.23
**Discharge GCS**						
3–8	1 (33.3)	2 (1.9)	0.14	0 (0.0)	2 (18.2)	0.01*
9–12	0 (0.0)	2 (1.9)		1 (1.5)	1 (9.1)	
13–15	2 (66.7)	100 (96.2)		68 (98.5)	8 (72.3)	

**GCS**: Glasgow Coma Scale; **SD**: standard deviation; **SBP**: systolic blood pressure; **SAH**: subarachnoid haemorrhage; ^**†**^ median (range/interquartile range);

* statistically significant at p = 0.05

^a^ Excludes the 4 arteriovenous malformations

^b^ Median (range/interquartile range)

**Fig 2:**
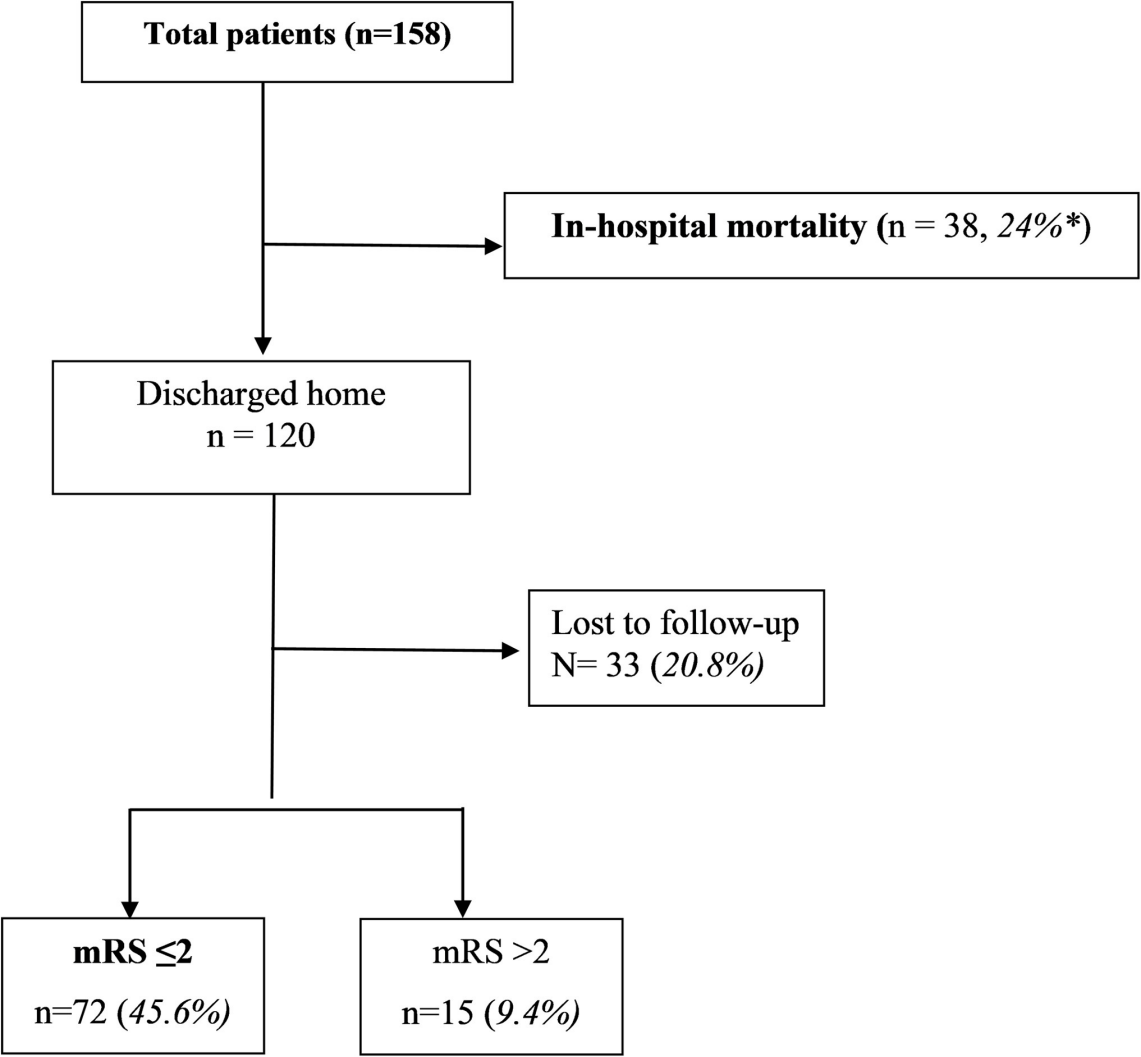
Pictorial representation of outcomes of SAH patients in our study. * percentages of all patients included in the study.

^c^ Includes only 87 patients/next of kin who could be contacted; 33 of the discharged patients were lost to follow-up (Fig 2)

Patients were managed either conservatively (79%, n = 124) or with surgical clipping (n = 27, 17.2%) after presenting to hospital 4 median days (interquartile range [IQR], 5 days) after the ictus. Six patients were transferred to other hospitals for coiling (3.8%), as this was not available in the study hospitals. Both public and private hospitals had delays in initiating definitive treatment, but the delays were significantly longer in public than private hospitals (median days from ictus to clipping: 19 days vs. 5.5 days, p = 0.006) (Table 1).

### Overall mortality and functional outcomes

The overall in-hospital mortality rate was 24.1% (n = 38) and the 1-month post-ictus mortality rate was 26.6% (n = 42). An almost equal number of males and females died in hospital [mean 6 days (Range, 4–15 days)]. Patients aged ≥50 years had a significantly higher in-hospital mortality rate than those aged <50 years (64.9% vs. 35.1%; p = 0.03). The median length of hospital stay was 8 days (IQR, 4–21) for non-survivors and 12 days (IQR, 8–17) for survivors (Table 2). Severity of ictus at admission was significantly associated to in-hospital mortality.

Assessment of functional outcomes targeted the 120 (75.9%) patients who were discharged. However, only 87 (72.5% of discharged patients, 55% of total patients) patients or next of kin could be reached via telephone. At follow up- six months to nine years after ictus, most patients discharged from hospital had favourable functional outcomes at follow up (n = 72; 82.8%, 95% CI: 74.7%–90.9%), with 17.2% (95% CI: 9.1%–25.3%) having unfavourable functional outcomes. This represented 45.6% of all patients who presented alive having favourable functional outcomes while 33.5% either died or were dependent and 21% were lost to follow-up (Fig 2).

A majority of patients with favourable functional outcomes were: female (n = 37, 51.4%), younger than 50 years (n = 40, 55.6%), had significantly lower average systolic blood pressure at admission (142 mmHg, SD: 27 mmHg) and a discharge GCS of 13–15 and had positive angiogram (n = 27, 38.6%). There were no significant differences in the functional outcomes between public and private hospitals (p = 0.53) (Table 2). Sex, admission systolic blood pressure, admission and discharge clinical status, presence of hydrocephalus and angiography status were significantly associated with functional outcomes as shown in Table 2 above.

### Outcomes by SAH type

Among 56 aneurysmal SAH patients, 26 (55%) were actively treated by microsurgical aneurysmal clipping and 25 (44.6%) were conservatively treated. Six patients (one non-aneurysmal and five aneurysmal) were transferred out of the study hospitals and treatment data was subsequently unavailable. Of the 26 aneurysmal SAH patients treated, 17 (65.4%) were <50 years of age, 14 (53.8% were in private hospitals, and 23 (88.5%) had a GCS of 13–15. The 25 conservatively managed aneurysmal SAH patients were mostly female, older [≥50 years], with longer median days from ictus to presentation. More patients were definitively treated in private than in public hospitals (78% vs. 36%, p = 0.005). A majority of aneurysmal SAH patients who were treated survived (n = 24, 92.3%) and had favourable functional outcomes (n = 18, 75%) (Table 3).

**Table 3:** Clinical, demographic and outcome data in 51 aneurysmal SAH ^b^ patients with and without definitive treatment.

Characteristics	Treated (n, %)	Untreated (n, %)	p-value ^c^
Number of patients	26 (55.4)	25 (44.6)	
**Hospital type**			
Public	12 (46.2)	21 (84.0)	0.005*
Private	14 (53.8)	4 (16.0)	
**Sex**			
Male	13 (50.0)	6 (24.0)	0.055
Female	13 (50.0)	19 (76.0)	
**Age**			
<50 years	17 (65.4)	9 (37.5)	0.049*
≥50 years	9 (34.6)	15 (62.5)	
**Admission GCS**			
9–12	3 (11.5)	7 (29.2)	0.164
13–15	23 (88.5)	17 (70.8)	
Length of hospital stay, days ^a^ (for survivors)	14 (10.5–24)	13.5 (6.5–24.5)	0.04*
Ictus to presentation, days ^a^	3 (1–5)	3 (1.5–7.5)	0.78
**In-hospital Mortality**			
Alive	24 (92.3)	20 (80.0)	0.248
Dead	2 (7.7)	5 (20.0)	
**Functional Outcomes**			
Favourable	18 (75.0)	9 (45.0)	
Unfavourable	2 (8.3)	4 (20.0)	0.139
Dead/ Lost to follow-up	4 (16.7)	7 (35.0)	

**GCS**: Glasgow Coma Scale;

^a^ median (range/interquartile range);

^b^ Patients transferred to other hospitals excluded;

^c^ Association between the characteristics and the treatment status of aneurysmal SAH patients;

* statistically significant at p = 0.05

Thirty-one SAH patients in our study (19.6%) had a negative cerebral angiogram; a majority of whom were younger than 50 years (55%) and had a GCS 13–15 at admission (80%). Only one of these patients died in the hospital (3.2%). Of those discharged home, twenty patients (65%) were reached and all of them had favourable outcomes. This group of patents had the best outcomes- mortality and functional outcomes- of all three groups based on angiography status (Tables 2 and 4 below).

**Table 4:** Clinical, demographic and outcome data in 158 patients based on SAH type.

Factors	Angiogram Status			p-value
	Not Done (n, %)	Positive (n, %) ^a^	Negative (n, %)	
Number of patients	67 (42.4)	60 (38.0)	31 (19.6)	
Age [mean (SD)]	51.5 (18.2)	46.5 (13.5)	46.4 (14.2)	
**Hospital type**				
Public	62 (92.5)	37 (61.7)	22 (71.0)	<0.01*
Private	5 (7.5)	23 (38.3)	9 (29.0)	
**Sex**				
Male	30 (44.8)	22 (36.7)	15 (48.4)	0.49
Female	37 (55.2)	38 (63.3)	16 (51.6)	
**Age**				
<50 years	17 (54.8)	34 (57.6)	17 (54.8)	0.25
≥50 years	14 (45.2)	25 (42.4)	14 (45.2)	
**Admission GCS**				
3–8	14 (21.2)	0 (0.0)	4 (13.3)	
9–12	16 (24.2)	11 (18.6)	2 (6.7)	<0.01*
13–15	36 (54.6)	48 (81.4)	24 (80.0)	
Length of hospital stay, days^b^ (for survivors)	9.5 (5–16)	14 (8–24)	12.5 (5–15)	<0.01*
Ictus to presentation, days^b^	4 (2.5–7)	3 (1–7)	3 (1–5)	<0.01*
**In-hospital Mortality**				
Dead	30 (44.8)	7 (11.7)	1 (3.2)	<0.01*
Alive	37 (55.2)	53 (88.3)	30 (96.8)	
**Functional Outcomes**				
Favourable	23 (34.3)	29 (48.3)	20 (64.5)	
Unfavourable	7 (10.5)	8 (13.3)	0 (0.0)	0.02*
Dead/ Lost to follow-up	37 (55.2)	23 (38.3)	11 (35.5)	
**Treatment**				
Conservative	66 (98.5)	29 (48.3)	29 (96.7)	<0.01*
Clip	1 (1.5)**	27 (43.3)	0 (0.0)	
Referral	0 (0.0)	5 (8.3)	1 (3.3)	

**GCS**: Glasgow Coma Scale; **SD**: standard deviation;

* statistically significant at p = 0.05;

^a^ Includes 4 arteriovenous malformations

^b^ Median (range/interquartile range)

**For one of the patients who underwent clipping, angiogram data was not recorded.

## Discussion

This study aimed to determine the mortality and functional outcomes of patients with spontaneous subarachnoid haemorrhage (SAH) in Kenya. We recorded 158 SAH cases over the nine-year study period. This low number of SAH cases could be explained by the retrospective nature of the study and possibly a large number of undiagnosed hence unreported SAH cases resulting from a lack of neuroimaging and angiographic diagnostic tools for SAH as seen in this study. This is also evidenced by the high proportion of autopsy identified SAH cases in unexplained deaths in Kenya [11]. Moreover, for SAH patients who eventually get diagnosed, record keeping in our region is deficient [18].

Our study found a relatively high overall mortality rate among patients with SAH of all types, with in-hospital and 1-month mortality rates of 24% and 26.6%, respectively. In-hospital mortality was highest for patients without angiography (44.8%) and least in the angiogram negative group (3.2%). The overall mortality was higher compared to other studies including aneurysmal and non-aneurysmal studies in the UK, Ireland and US where the rates reported were between 11–18% [3,19]. This might result from delayed presentation as shown by protracted ictus to presentation times (mean 4 days), delayed treatment (mean 9 days) and high numbers of untreated patients seen in this study.

For aneurysmal SAH patients, the in-hospital mortality rate was 11.7% which was much lower compared with Saudi Arabia (15%) [20], in Morocco (18%) [14], and Nairobi, Kenya (30%) [15]. This should however be interpreted in light of the limitations of this study and may result from evident treatment bias. Conservatively managed aneurysmal SAH patients had in-hospital mortality rates nearly three times that of surgically clipped patients (20% and 7.7%). Surgically clipped patients also had nearly two-fold favourable outcomes as compared to the conservatively managed patients (75% and 45%). This is supported by existing literature that indicate actively managed patients have lower mortality rates and better functional outcomes compared with conservatively managed patients [21,22].

Compared with angiogram-negative SAH patients however, patients with aneurysmal SAH had an in-hospital mortality rate four times higher (11.7% compared to 3.2%). This is comparable with Elhadi *et al*.’s US study that reported zero in-hospital mortality, with 64% of patients having a complete recovery at discharge and a short hospital stay (mean 9.5 days, range 2–27 days) for non-aneurysmal SAH patients [23]. The length of stay in our study was however more protracted, with a mean of 12.5 days (range 5–15 days) for angiogram negative SAH patients in our study.

Overall, majority of patients discharged from hospital who were followed up in our study had favourable outcomes (83%) six or more months after ictus; this however represents only 45.6% of all the patients who presented alive; much lower than that recorded in other studies (50–60%) in different regions [19,21]. Only 58% of patients had angiographies in our study compared to over 90% in the high-income settings [22]; and subsequently, only 55% of the aneurysmal SAH patients in our study were definitively treated (by surgical clipping); while 45% were untreated. This is unlike the 17–38% untreated cases reported in other studies [22] and the large number of untreated patients may explain the worse outcomes reported in our study. Additionally, for SAH patients who were actively treated, treatment was instituted 9 days from the ictus compared with 2.7 days in the UK [21]. The delay in treatment was significantly longer in the public hospital where access to neurosurgical services and quality of care is low. Public hospitals have limited neuro-intensive care, low access to neurosurgical operation space, and no endovascular services [24] while private hospitals have better neurosurgical infrastructure but are costly for a majority of Kenyans [25]. Hence, the difference in outcomes between public and private hospitals, and between Kenya and high-resourced countries.

Although there are limited comparable studies in Africa, a 17-year retrospective review of patients with intracranial aneurysms in Morocco reported a 64.5% favourable outcomes [14]. The differences in favourable functional outcomes could be explained by the differences in inclusion criteria as the Moroccan study only included treated patients, as all conservatively managed patients were lost to follow up. Considering only definitively-treated patients in our study, nearly two-thirds (75%) of all surgically clipped patients had favourable functional outcomes; which would be comparable to the Moroccan study, and similar retrospective studies in Senegal and South Africa that reported a 64.7% and 66% favourable outcomes after surgical clipping [13,26].

Our study had a higher number of angiogram-negative SAH cases (34%) compared with other studies [27,28]. All of them experienced favourable outcomes comparable to other studies (83% [27] and 94% [29]). While this may be intriguing, it is noteworthy that none of these angiogram negative SAH patients had a repeat angiogram to confirm the non-aneurysmal SAH. The patients’ outcomes in our study were associated with the SAH type, severity of initial bleed (admission GCS), admission blood pressure, discharge GCS, presence of hydrocephalus and sex after univariate analysis. These factors have been shown to affect patient survival and functional outcomes after SAH in other studies [19,21,30–32].

### Limitations

To our knowledge, this is the first study in Kenya focusing on outcomes for patients after SAH. This study was conducted in three major public and private hospitals in Nairobi, with a relatively large representative sample of all SAH cases in Kenya. Therefore, our findings could be generalisable to Kenya. This study also used a standardised definition for SAH and a pretested checklist to avoid misclassification bias and allow for comparability of the findings and forms a basis for future large-scale prospective studies. However, despite its strengths, the study had some limitations.

First, the study was retrospective and therefore has potential for information bias (e.g. recall and misclassification bias). This was mitigated by using two data sources (medical files and patient/next of kin interviews) but was not fully addressed because some patients were unreachable. In addition, some data were missing, such as patients’ date of demise. There was also a risk of misclassification bias because the study relied on the accuracy and reliability of diagnoses made by other medical doctors. During the telephone interviews, there was also a potential risk of responder bias.

Second, some of the secondary outcomes could not be assessed because of missing data, which still is a common problem in our region due to reliance on paper-based records [18], such as cases of re-bleeding and delayed ischaemic neurological deficits, the incidence of symptomatic vasospasm, readmissions and mortality from other causes. Subsequently, there was a high rate of loss of follow up, owing to the retrospective nature of the study and the missing data. As such, some conclusions couldn’t be concretely made.

Third, lack of local hospital guidelines/protocols on SAH management means that most cases are totally determined by the attending physician, with minimal or no consultation. As such, more younger patients with good admission clinical status and especially in private hospitals were definitively treated indicating possible treatment bias. Lastly, due to lack of angiograms, it was difficult to characterize a significant number of SAH cases adequately. This was further hampered by the retrospective nature of the study.

## Conclusion

In-hospital and 1-month mortality rates for SAH are high in Kenya, and functional outcomes are generally low. For patients who reach neurosurgical centres and are actively managed however, favourable functional outcomes are comparable to other centres; although given the limitations of this study including treatment bias, this needs to be further studied.

As one of the first studies on the outcomes of patients with SAH in sub-Saharan Africa, this study highlights the disparity in outcomes in SAH patients between low- and high-resourced regions. This study forms a basis for further prospective studies to investigate other secondary outcomes of SAH, such as readmissions and re-bleeding.

## Supporting information

S1 File Subarachnoid_hemorrhage_Kenya data. (DTA)

S1 Table Sample characteristics by hospital type. (DOCX)
