# Supplementary material for: Mortality and functional outcomes after a spontaneous subarachnoid haemorrhage: A retrospective multicentre cross-sectional study in Kenya
Source: PLoS One. 2019 Jun 12;14(6):e0217832. doi: 10.1371/journal.pone.0217832 (PMC6561561; doi:10.1371/journal.pone.0217832)
Supplement: S1 Table — (DOCX) [file pone.0217832.s002.docx]

**S1 Table. Sample characteristics by hospital type**

|  | Variables | Private | Public | Total | p-value |  |
| --- | --- | --- | --- | --- | --- | --- |
|  | Number of Patients | 37 (23.4) | 121 (76.6) | 158 |  |  |
| Demographic and  clinical characteristics | **Sex** |  | | | | |
|  | Male | 20 (54.1) | 47 (38.8) | 67 (42.4) | 0.101 |  |
|  | Female | 17 (45.9) | 74 (61.2) | 91 (57.6) |  |  |
|  | **Age** |  | | | | |
|  | <50 years | 19 (51.4) | 61 (50.4) | 80 (50.6) | 0.268 |  |
|  | ≥50 years | 17 (46.0) | 60 (49.6) | 77 (48.7) |  |  |
|  | Missing | 1 (2.7) | 0 (0.0) | 1 (0.6) |  |  |
|  | History of hypertension |  |  |  |  |  |
|  | Yes | 14 (37.8) | 65 (53.7) | 79 (50.0) |  |  |
|  | No | 20 (54.1) | 54 (44.6) | 74 (46.8) | 0.051 |  |
|  | Missing | 3 (8.1) | 2 (1.7) | 5 (3.2) |  |  |
|  | **Tobacco use** |  |  |  |  |  |
|  | Yes | 4 (10.8) | 14 (11.6) | 18 (11.4) |  |  |
|  | No | 22 (59.5) | 87 (71.9) | 109 (69.0) | 0.208 |  |
|  | Missing | 11 (29.7) | 20 (16.5) | 31 (19.6) |  |  |
|  | **Alcohol use** |  |  |  |  |  |
|  | Yes | 12 (32.4) | 20 (16.5) | 32 (20.3) | 0.009 |  |
|  | No | 14 (37.8) | 80 (66.1) | 94 (59.5) |  |  |
|  | Missing | 11 (29.7) | 21 (17.4) | 32 (20.3) |  |  |
|  | **Depressed consciousness** |  |  |  |  |  |
|  | Yes | 19 (51.4) | 64 (52.9) | 83 (52.5) | 0.796 |  |
|  | No | 18 (48.6) | 55 (45.4) | 73 (46.2) |  |  |
|  | Missing | 0 (0.0) | 2 (1.7) | 2 (1.3) |  |  |
|  | **Seizures** |  |  |  |  |  |
|  | Yes | 8 (21.6) | 26 (21.5) | 34 (21.5) |  |  |
|  | No | 20 (54.1) | 93 (76.9) | 122 (77.2) | >0.99 |  |
|  | Missing | 0 (0.0) | 2 (1.7) | 2 (1.3) |  |  |
|  | **Headache** |  |  |  |  |  |
|  | Yes | 32 (86.5) | 103 (85.1) | 135 (85.4) |  |  |
|  | No | 5 (13.5) | 16 (13.2) | 21 (13.3) | 0.992 |  |
|  | Missing | 0 (0.0) | 2 (1.7) | 2 (1.3) |  |  |
|  | **Neck pain or stiffness** |  |  |  |  |  |
|  | Yes | 11 (29.7) | 57 (47.1) | 68 (43.6) | 0.108 |  |
|  | No | 26 (70.3) | 62 (51.2) | 88 (55.7) |  |  |
|  | Missing | 3 (8.1) | 2 (1.7) | 2 (1.7) |  |  |
|  | **Focal neurological deficits** |  |  |  |  |  |
|  | Yes | 3 (8.1) | 21 (13.5) | 21 (13.5) |  |  |
|  | No | 34 (91.9) | 101 (83.5) | 135 (85.4) | 0.576 |  |
|  | Missing | 0 (0.0) | 2 (1.7) | 5 (3.2) |  |  |
|  | Ictus to presentation, days† (n=141) | 3 (1–5) | 4 (2–7) | 4 (2–7) | 0.126 |  |
|  | Admission SBP (n=152), mean (SD) | 152 (34.1) | 147.5 (28.1) | 148.5 (29.6) | 0.043* |  |
|  | **Admission GCS** |  | | | | |
|  | 3–8 | 3 (8.1) | 15 (12.4) | 18 (11.4) | 0.157 |  |
|  | 9–12 | 3 (8.1) | 26 (21.5) | 29 (18.4) |  |  |
|  | 13–15 | 30 (81.1) | 78 (64.5) | 108 (68.4) |  |  |
|  | Missing | 1 (2.7) | 2 (1.7) | 3 (1.9) |  |  |
| Radiological features | **Modified Fisher’s score** |  | | | | |
|  | 1 | 6 (16.2) | 0 (0.0) | 6 (3.8) |  |  |
|  | 2 | 3 (8.1) | 2 (1.7) | 5 (3.2) |  |  |
|  | 3 | 5 (13.5) | 8 (6.6) | 13 (8.2) | 0.000 |  |
|  | 4 | 7 (18.9) | 7 (5.8) | 14 (8.9) |  |  |
|  | Missing score | 16 (43.2) | 104 (86.0) | 120 (76.0) |  |  |
|  | **Hydrocephalus** |  |  |  |  |  |
|  | Yes | 11 (29.7) | 18 (14.9) | 29 (18.4) |  |  |
|  | No | 8 (21.6) | 53 (43.8) | 61 (38.6) | 0.023 |  |
|  | Missing | 18 (48.7) | 50 (41.3) | 68 (43.0) |  |  |
| Angiography | **Angiogram Status** |  |  |  |  |  |
|  | Positive | 23 (62.2) | 37 (30.6) | 60 (38.0) |  |  |
|  | Negative | 9 (24.3) | 22 (18.2) | 31 (19.6) | 0.215 |  |
|  | Not Done | 5 (13.5) | 62 (51.2) | 67 (42.4) |  |  |
|  | **Angiogram type (n=91)** |  | | | | |
|  | Computed tomography | 26 (81.3) | 15 (25.4) | 41 (45.1) | <0.001* |  |
|  | Magnetic resonance | 1 (3.1) | 6 (10.2) | 7 (7.7) |  |  |
|  | Digital subtraction | 5 (15.6) | 38 (64.4) | 43 (47.2) |  |  |
|  | **Aneurysm location (n=56)** |  | | | | |
|  | Anterior (ACA/AcoA) | 11 (47.8) | 15 (39.5) | 24 (42.9) | 0.832 |  |
|  | Middle (MCA) | 6 (26.1) | 8 (21.1) | 11 (19.6) |  |  |
|  | Internal (ICA/PcoA) | 4 (17.4) | 7 (18.4) | 11 (19.6) |  |  |
|  | Posterior (PCA/VA) | 0 (0.0) | 2 (5.3) | 1 (0.02) |  |  |
|  | Multiple | 2 (8.7) | 6 (15.8) | 9 (16.1) |  |  |
|  | **Aneurysm size (n=56)** |  | | | | |
|  | Small | 6 (26.1) | 1 (3.0) | 7 (12.5) | 0.350 |  |
|  | Medium | 7 (30.4) | 1 (3.0) | 8 (14.3) |  |  |
|  | Large | 0 (0.0) | 1 (3.0) | 1 (1.8) |  |  |
|  | Missing | 10 (43.5) | 30 (90.9) | 40 (71.4) |  |  |
| Management | **Mode of treatment** |  | | | | |
|  | Conservative | 17 (46.0) | 108 (89.3) | 125 (79.1) | <0.001* |  |
|  | Clip | 14 (37.8) | 13 (10.7) | 27 (17.1) |  |  |
|  | Referral (for coiling) | 6 (16.2) | 0 (0.0) | 6 (3.8) |  |  |
|  | Days from ictus to clipping (n=24) | 5.5 (2–9) | 19 (11–58) | 9 (4–20.5) | 0.006* |  |
|  | Length of hospital stay (n=156) | 9 (7–15) | 11 (5–21) | 10.5 (5–21) | 0.250 |  |
|  | **Discharge GCS** |  | | | | |
|  | 3–8 | 2 (5.4) | 1 (0.8) | 3 (1.9) | 0.02 |  |
|  | 9–12 | 0 (0.0) | 2 (1.6) | 2 (1.3) |  |  |
|  | 13–15 | 29 (78.4) | 73 (60.3) | 102 (64.6) |  |  |
|  | Missing | 6 (16.2) | 45 (37.2) | 51 (32.3) |  |  |
